# Glypican-3 induces a mesenchymal to epithelial transition in human breast cancer cells

**DOI:** 10.18632/oncotarget.11107

**Published:** 2016-08-06

**Authors:** Lilian Fedra Castillo, Rocío Tascón, María Amparo Lago Huvelle, Gisela Novack, María Candelaria Llorens, Ancely Ferreira dos Santos, Jorge Shortrede, Ana María Cabanillas, Elisa Bal de Kier Joffé, Leticia Labriola, María Giselle Peters

**Affiliations:** ^1^ Universidad de Buenos Aires, Instituto de Oncología “Ángel H. Roffo”, Area Investigación, Buenos Aires, Argentina; ^2^ Universidad de Buenos Aires, CONICET, Instituto de Química Biológica de la Facultad de Ciencias Exactas y Naturales (IQUIBICEN), Facultad de Ciencias Exactas y Naturales, Buenos Aires, Argentina; ^3^ Departamento de Bioquímica Clínica, Facultad de Ciencias Químicas, Universidad Nacional de Córdoba and Centro de Investigaciones en Bioquímica Clínica e Inmunología (CIBICI-CONICET), Córdoba, Argentina; ^4^ Biochemistry Department, Chemistry Institute, University of São Paulo, São Paulo, Brazil

**Keywords:** breast cancer, glypican-3, epithelial-mesenchymal transition, invasion, metastasis

## Abstract

Breast cancer is the disease with the highest impact on global health, being metastasis the main cause of death. To metastasize, carcinoma cells must reactivate a latent program called epithelial-mesenchymal transition (EMT), through which epithelial cancer cells acquire mesenchymal-like traits.

Glypican-3 (GPC3), a proteoglycan involved in the regulation of proliferation and survival, has been associated with cancer. In this study we observed that the expression of GPC3 is opposite to the invasive/metastatic ability of Hs578T, MDA-MB231, ZR-75-1 and MCF-7 human breast cancer cell lines. GPC3 silencing activated growth, cell death resistance, migration, and invasive/metastatic capacity of MCF-7 cancer cells, while GPC3 overexpression inhibited these properties in MDA-MB231 tumor cell line. Moreover, silencing of GPC3 deepened the MCF-7 breast cancer cells mesenchymal characteristics, decreasing the expression of the epithelial marker E-Cadherin. On the other side, GPC3 overexpression induced the mesenchymal-epithelial transition (MET) of MDA-MB231 breast cancer cells, which re-expressed E-Cadherin and reduced the expression of vimentin and N-Cadherin. While GPC3 inhibited the canonical Wnt/β-Catenin pathway in the breast cancer cells, this inhibition did not have effect on E-Cadherin expression. We demonstrated that the transcriptional repressor of E-Cadherin - ZEB1 - is upregulated in GPC3 silenced MCF-7 cells, while it is downregulated when GPC3 was overexpressed in MDA-MB231 cells. We presented experimental evidences showing that GPC3 induces the E-Cadherin re-expression in MDA-MB231 cells through the downregulation of ZEB1.

Our data indicate that GPC3 is an important regulator of EMT in breast cancer, and a potential target for procedures against breast cancer metastasis.

## INTRODUCTION

Breast cancer is the leading cause of female mortality due to malignant diseases worldwide [1]. Despite recent major advances in the understanding of the mechanisms of breast cancer progression and in the development of novel therapeutic modalities, metastatic disease still remains the most critical condition limiting patient survival [2]. The chance of five year survival following diagnosis falls from >90% for localized disease to <20% once metastasis has occurred.

Metastasis is defined as the formation of progressively growing secondary tumor foci at sites discontinuous from the primary lesion. To metastasize, carcinoma cells must reactivate a latent embryonic program called epithelial-mesenchymal transition (EMT) [3]. EMT marks the first step of the “metastasis cascade”, where epithelial cells of the primary tumor acquire mesenchymal-like traits. This way, epithelial cells lose their cell-cell adhesion and apical-basal polarity and change to a fibroblastic phenotype, modulate the organization of their cytoskeleton and gain the ability to migrate individually and invade basement membrane and blood vessels. Upon intravasation these cells stay in the bloodstream as circulating tumor cells, until they exit at some distant organs to initiate their colonization [3, 4]. Epithelial cells that undergo the EMT lose epithelial markers expression - such as E-Cadherin - while they acquire mesenchymal ones, like vimentin and N-Cadherin [5].

Different signaling pathways like TGF-β, EGF, HGF, Notch, FGF, Wnt, and IGFs [6], as well as mechanical factors such as extracellular matrix density [7], control EMT. These signals activate one of the EMT-inducing transcription factors – TWIST1, SNAIL1, SNAIL2 (SLUG), ZEB1, ZEB2 (SIP1), Brachyury, Goosecoid, SIX1, and PRRX1 – that directly or indirectly repress the hallmark of epithelial phenotype, E-Cadherin expression. On the other hand, EMT can be inhibited by p53, mesenchymal-epithelial (MET)-inducing transcription factors such as GRHL2 and ELF5, and microRNA (miR) families like miR-200 and miR-34 [8].

Glypican-3 (GPC3) is a member of the heparan sulphate proteoglycan family that is attached to the cell surface by a glycosylphosphatidylinositol (GPI) anchor. Expression of GPC3 is substantial in trophoblasts and a number of embryonic tissues [9]. Modulation of its levels in a stage- and tissue specific manner has already been shown, suggesting an involvement in morphogenesis [10, 11]. It was reported that in the adult, GPC3 is expressed only in a few tissues including mesothelium, and the ovarian and mammary epithelia [12, 13]. In spite of the fact that proteoglycans, which are strategically located on the surface of the extracellular matrix (ECM) cells and in the pericellular matrix, are a key component in stromal-epithelial interactions and signaling [14], only a few scientists have addressed their role under normal conditions in the healthy breast [15].

There are several studies which have linked GPC3 with cancer [16]. In this regard, GPC3 overexpression has been shown in Wilms' tumor [17], hepatocellular carcinoma (HCC) [18–20], yolk sac tumor and clear cell ovarian carcinoma [21]. In contrast, GPC3 expression is downregulated in lung adenocarcinoma [22], cell clear renal carcinoma [23], mesothelioma, ovarian [24, 25] and gastric cancer [26]. Regarding breast cancer, we have recently performed comparative studies of GPC3 expression, indicating lower GPC3 levels in tumors as compared to peritumor tissues [27]. In summary, depending on the tissue, GPC3 displays a very different pattern of expression during tumor progression. In tumors originated from tissues that only express GPC3 in the embryonic stage, the expression of this glypican tends to reappear upon malignant transformation. On the other hand, in cancers originated from tissues that are GPC3-positive in the adulthood, the expression of GPC3 is reduced during tumor progression. It is speculated that this tissue-specific differences are due to the fact that GPC3 is regulating different growth and survival factors in each tissue [16]. In this regard, we previously showed that the ectopic expression of GPC3 in the LM3 murine mammary adenocarcinoma cell line was able to inhibit invasion and metastasis [28]. Although the GPC3 signaling mechanism is not completely elucidated, we found that GPC3 re-expressing murine cells displayed an inhibition of the canonical Wnt signaling as well as an activation of the non-canonical Wnt/PCP pathway [29]. We also demonstrated that GPC3 re-expression inhibited the PI3K/AKT pathway and stimulated the p38 MAPK cascade [30].

In view of the clinical and translational usefulness of GPC3, in the current study we generated and characterized engineered human breast cancer cells to evaluate the role of GPC3 on human mammary tumor progression. We present *in vitro* and *in vivo* experimental evidence supporting the hypothesis that GPC3 has a protective role against human breast cancer progression. Furthermore, in this work we demonstrate that GPC3 induces MET. GPC3 expressing cells exhibit an epithelial phenotype, change their cytoskeleton organization, reduce their migration and clonogenic abilities, are more susceptible to cell death, exhibit higher homotypic adhesion, express epithelial markers while lose mesenchymal ones and are less invasive/metastatic. We showed that when human mammary tumor cells express GPC3, the canonical Wnt pathway is inhibited, the transcription factors ZEB1 is downregulated and the key marker of epithelial phenotype E-Cadherin is upregulated. So, cell-cell contacts are stabilized and cell detachment is diminished, thereby inhibiting the invasive and metastatic capacity of breast tumors.

## RESULTS

### Generation of engineered breast cancer cells

#### Analysis of GPC3 expression in breast cancer human cell lines

**Figure 1:**
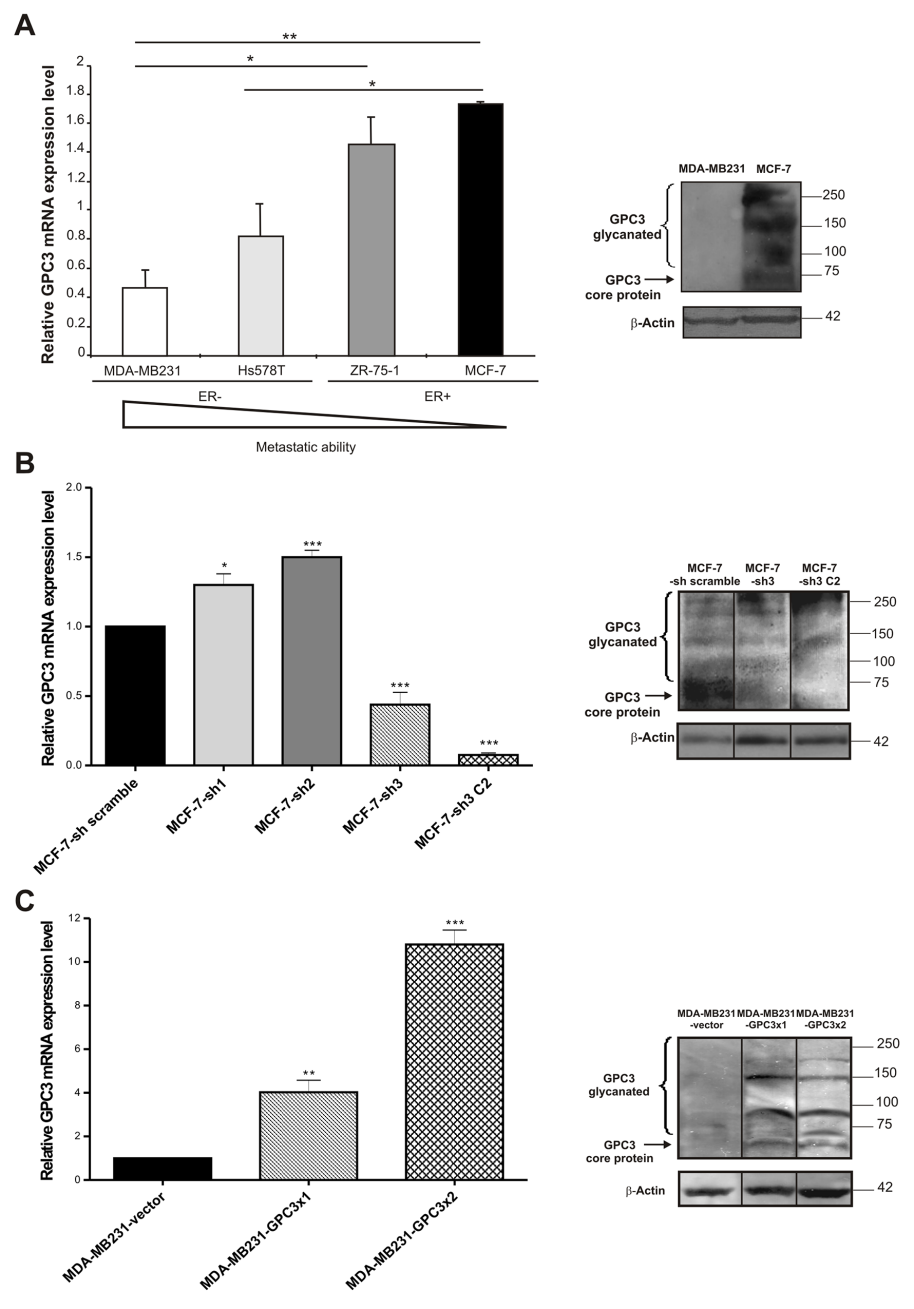
GPC3 expression in breast cancer cell lines **A, B, C.** *Left panel*: GPC3 mRNA expression levels were identified by qRT-PCR analysis of MDA-MB231, Hs578T, ZR-75-1 and MCF-7 (A), MCF-7-scramble, MCF-7-sh1, MCF-7-sh2, MCF-7-sh3 and MCF-7-sh3 C2 (B), MDA-MB231-vector, MDA-MB231-GPC3×1 and MDA-MB231-GPC3×2 (C) cells. GAPDH was used as control. Values are expressed as mean ± SD. The data are representative of three independent experiments. (*p<0.05, **p<0.01, ***p<0.001 ANOVA, Tuckey test). *Right panel:* WB analysis was used to determine GPC3 protein expression in MDA-MB231 and MCF-7 (A), MCF-7-sh scramble, MCF-7-sh3 and MCF-7-sh3 C2 (B), MDA-MB231-vector, MDA-MB231-GPC3×1 and MDA-MB231-GPC3×2 (C) cells. β-Actin was used as an internal control. The arrow indicates the GPC3 protein core and the bracket indicates the glycanated fragments. Black lines highlight spliced lanes within a gel. Numbers on the right represent molecular mass (kDa).

To study whether human breast cancer cell lines express GPC3, a qRT-PCR analysis was performed. Two groups of cell lines representing different stages of the disease were chosen (Table 1). Our results suggested that the GPC3 mRNA expression level was opposite to the invasive and metastatic abilities of the studied human cell lines. The invasive and metastatic Hs578T and MDA-MB231 cell lines expressed lower GPC3 mRNA levels than the poorly metastatic ZR-75-1 and MCF-7 cell lines (p<0.05 Hs578T vs. MCF-7 and MDA-MB231 vs. ZR-75-1, p<0.01 MDA-MB231 vs. MCF-7, Figure 1A, left panel). In addition, 2.77 times higher levels of GPC3 mRNA were found in MCF10A mammary normal-like cells than in MCF-7 cells (p<0.01, data not shown). We confirmed the GPC3 expression at protein level by WB in selected cell lines (MCF-7 and MDA-MB231) (Figure 1A, right panel).

**Table 1:** Characteristics of the human breast cancer cell lines

Cell Line	Histopathological Type	Origin	ER	PR	Invasive Potential	Metastatic Potential
MDA-MB231	Invasive ductal carcinoma	Metastasis (pleural effusion)	-	-	high	moderate/high
HS578T	Carcinosarcoma	Primary Tumor	-	-	high	High
ZR-75-1	Invasive ductal carcinoma	Metastasis (ascites fluid)	+	+	moderate/low	Null
MCF-7	Invasive ductal carcinoma	Metastasis (pleural effusion)	+	+	low	Null

ER: estrogen receptor; PR: progesterone receptor; + Positive; - Negative

Table based on previously published data [41].

#### Silencing of GPC3 expression

Since MCF-7 was the cell line that expressed the highest levels of GPC3 (Figure 1A), we decided to inhibit GPC3 expression by means of shRNA technology. As was described in Materials and Methods, we used three shRNA sequences specific for GPC3 (designed as sh1, sh2 and sh3), as well as a shRNA scramble and a shRNA GAPDH sequences as controls (Table 2). After selection with G418, antibiotic-resistant colonies were screened for GPC3 expression by qRT-PCR (Figure 1B, left panel). Since the greatest silencing was obtained with the sh3 construction, we decided to clone this cell subline (called MCF-7-sh3). We selected one clone, named MCF-7-sh3 C2, with 95% of reduction in the GPC3 mRNA expression levels (p<0.001 MCF-7-sh3 and MCF-7 sh3 C2 vs. MCF-7-sh scramble, Figure 1B, left panel). GPC3 depletion was also validated at protein level by WB (Figure 1B, right panel).

**Table 2:** shRNA specific sequences used to silence GPC3 expression in MCF-7 cell line

Name	Target sequence	ΔG Hairpin (Kcal/mol)	Loop	Tm (°C)
sh1	GGCTCTGAATCTTGGAATTGA	−35.8	7 nt	114
sh2	GCCGAAGAAGGGAACTAATTC	−38.0	7 nt	113
sh3	GGGACTGATGGTTAAACC	−35.6	7 nt	114
sh scramble	GTTCTCCGAACGTGTGTCACGT	−31.8	9 nt	121
sh GAPDH	GTATGACAACAGCCTCAAG	−29.0	7 nt	111

sh1: shRNA GPC3 1; sh2: shRNA GPC3 2; sh3: shRNA GPC3 3; sh scramble: shRNA scramble; sh GAPDH: shRNA GAPDH; nt: nucleotide

#### Overexpression of GPC3

Given that the MDA-MB231 malignant tumor cell line presented the lowest level of GPC3 (Figure 1A), we chose this cell line to overexpress GPC3. We performed one (MDA-MB231-GPC3×1) or two (MDA-MB231-GPC3×2) rounds of infection with lentivirus containing the GPC3 cDNA or with the empty vector (MDA-MB231-vector) as control. When we evaluated the expression of GPC3 using qRT-PCR, we detected a 5 fold increase of the GPC3 mRNA levels in MDA-MB231-GPC3×1 cells, whereas this increase was 10 times higher in the MDA-MB231-GPC3×2 cells (p<0.01 MDA-MB231-GPC3×1 vs. MDA-MB231-vector, p<0.001 MDA-MB231-GPC3×2 vs. MDA-MB231-vector, Figure 1C, left panel). Overexpression of GPC3 was also determined at the protein level (Figure 1C, right panel).

### Characterization of the breast cancer cells with genetically modified GPC3 expression

#### *In vitro* cell behavior

##### Cell morphology

**Figure 2:**
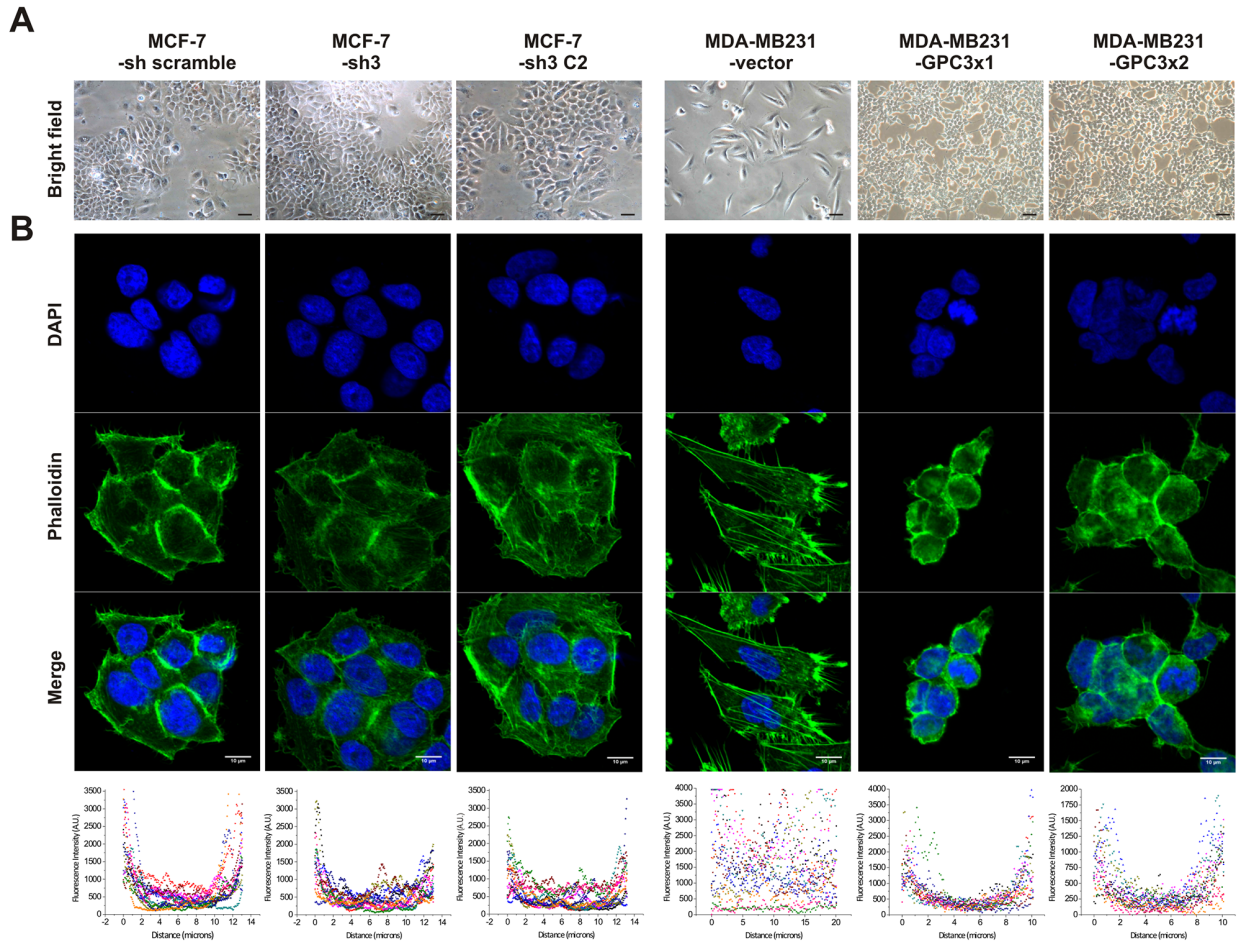
Effect of GPC3 on cell morphology and actin cytoskeleton organization **A.** Morphological characteristics of MCF-7 and MDA-MB231 cell sublines. Representative Bright Field (BF) images are shown (Magnification x200, scale bars 40 μm). **B.** Fluorescence microscope analysis of actin cytoskeleton in MCF-7 and MDA-MB231 cell sublines after phalloidin-FITC staining. Nuclei were stained with DAPI. Representative images are shown (Magnification x600, scale bars 10 μm). The scatter plots represent the quantification of fluorescence intensity across the lines of 12 cells of each group using ImageJ software.

Although MCF-7-sh3 and control cells were morphologically similar and grew as monolayer of epithelial polyhedral cells, we found that GPC3 overexpressing MDA-MB231 cells lost their fibroblast-like appearance, acquiring an epithelial morphology (Figure 2A).

To analyze in detail the morphological change induced by GPC3, F-Actin organization was examined using phalloidin-FITC staining. We processed the confocal images and generated a graphic depiction where the x-axis represented the distance across the cell and the y-axis symbolized the level of fluorescence. It was determined that, although the actin of MCF-7 cells appeared mainly in the cortical position as was expected for epithelial cells, GPC3 silencing induced the assembly of few F-Actin stress fibers (Figure 2B). In addition, although control cells showed large actin stress fibers, GPC3 overexpression in MDA-MB231 cells induced the loss of these fibers and the re-localization of actin mainly in a cortical cell distribution (Figure 2B).

##### Cell growth properties

**Figure 3:**
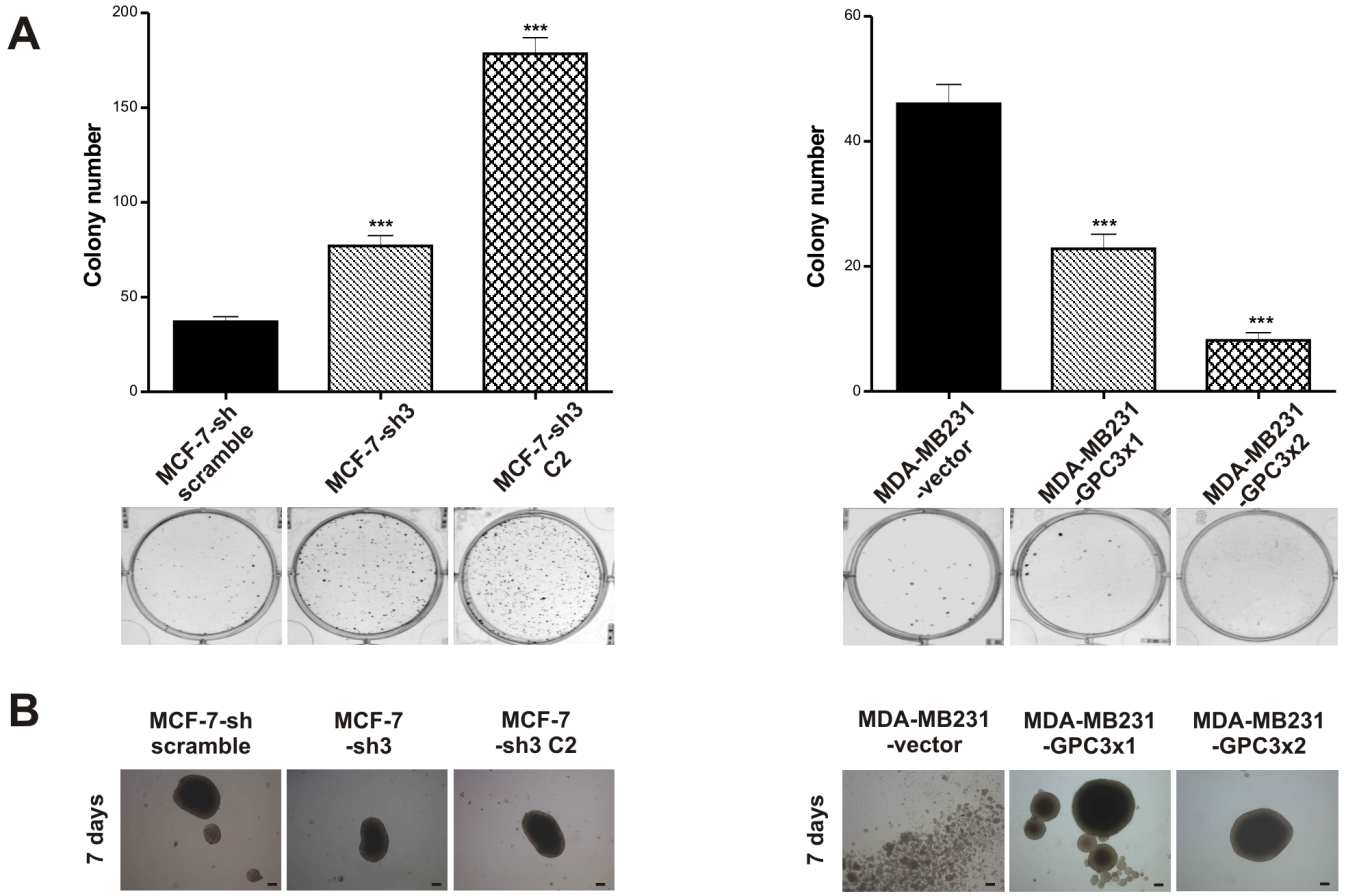
Effect of GPC3 on cell growth properties **A.** Clonogenic assays were employed to test the ability to grow at low density of MCF-7-sh scramble, MCF-7-sh3 and MCF-7-sh3 C2, as well as MDA-MB231-vector, MDA-MB231-GPC3×1 and MDA-MB231-GPC3×2 cells. Values are expressed as mean ± SD. The figure shows the results of one of three independent experiments. (***p<0.001 ANOVA, Tuckey test). **B.** The anchorage-independent 3D growth potential of MCF-7 and MDA-MB231 cell sublines was examined by the spheroids formation. Images were taken under inverted phase contrast microscope and are representative of two independent experiments (Magnification x40, scale bars 200 μm).

To investigate the ability of the engineered sublines to grow at low density, we performed clonogenic assays. The results showed that GPC3 knock down induced a significant increase in the clonogenicity of MCF-7 cells, while GPC3 overexpression suppressed MDA-MB231 clonogenic capacity (p<0.001 MCF-7-sh3 and MCF-7-sh3 C2 vs. MCF-7-sh scramble; p<0.001 MDA-MB231-GPC3×1 and MDA-MB231-GPC3×2 vs. MDA-MB231-vector, Figure 3A).

We also tested the anchorage-independent growth and checked that GPC3 silencing did not induce significant changes in the organization of MCF-7 spheroids (Figure 3B). Interestingly, although MDA-MB231 cells were unable to form spheroids, they acquired this ability when GPC3 was overexpressed (Figure 3B).

Overall, these data indicate that GPC3 suppressed low density growth as well as activated cell-cell adhesion and spheroids formation of the mammary cancer cells.

##### Susceptibility to cell death induction

To further investigate the functional role of GPC3 in breast cancer, the sensitivity of the different sublines to nutrient depletion was tested.

**Figure 4:**
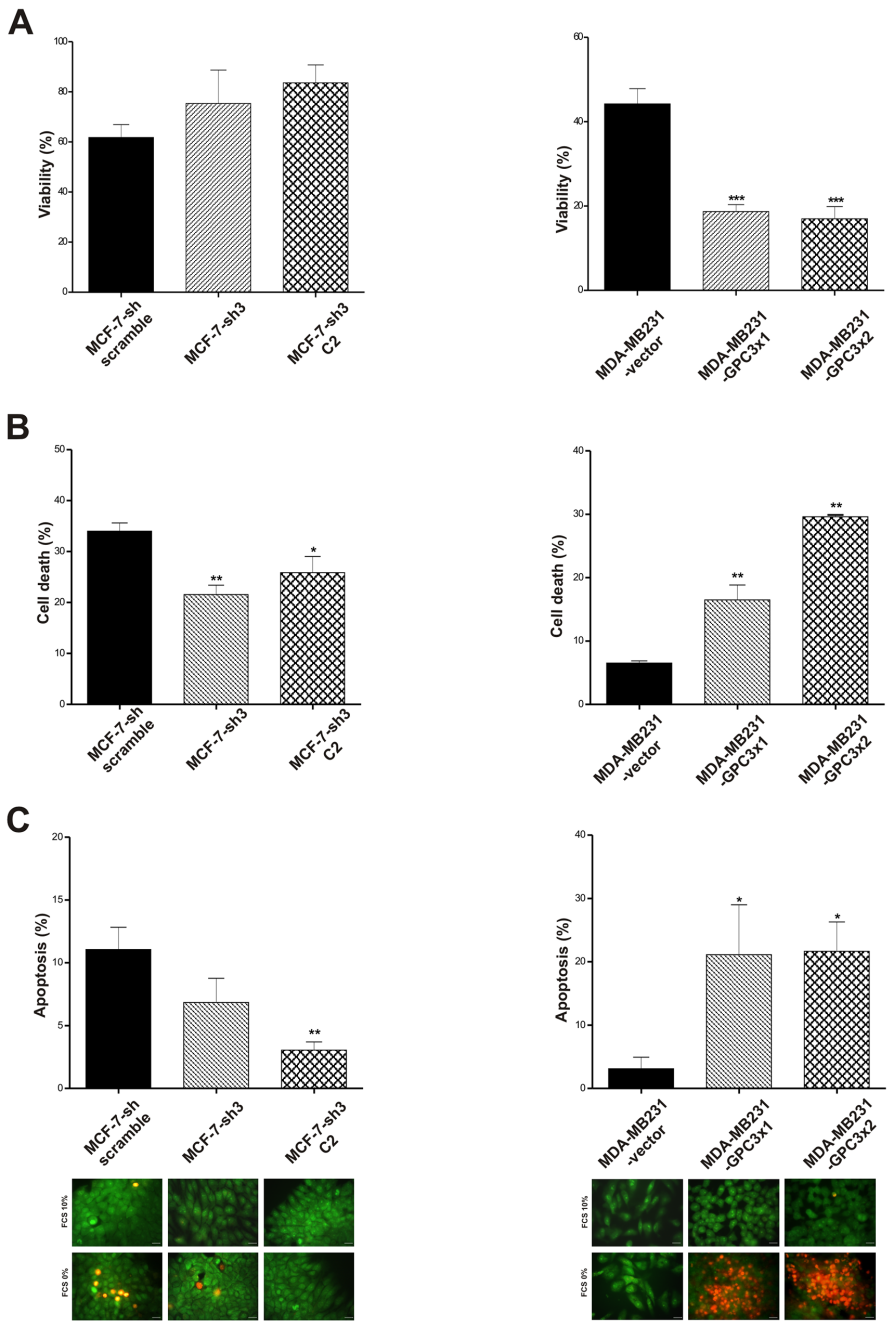
Effect of GPC3 on susceptibility to cell death **A.** After 72 h starving, the number of MCF-7-sh scramble, MCF-7-sh3 and MCF-7-sh3 C2, as well as of MDA-MB231-vector, MDA-MB231-GPC3×1 and MDA-MB231-GPC3×2 viable cells was evaluated by Trypan blue staining (*** p<0.001 ANOVA, Tuckey test). **B.** Starved MCF-7 and MDA-MB231 cell sublines were stained with propidium iodide / Höescht and the cell death rate was calculated (*p<0.05, **p<0.01 ANOVA, Dunnett test). **C.** Acridine orange / ethidium bromide staining was employed to evaluate apoptotic cell death. The number of apoptotic cells was recorded and plotted (*p<0.05, **p<0.01 ANOVA, Dunnett test). Characteristic images are showed (Magnification x400, scale bars 20 μm). A, B and C values are expressed as mean ± SD and data are representative of three independent experiments.

After 72 h of starving we found, through Trypan Blue vital staining, that GPC3 silencing induced an increase in the MCF-7 cells viability (Figure 4A). Similar results were obtained by Propidium Iodide / Höechst staining (p<0.05 MCF-7-sh3 C2 vs. MCF-7-sh scramble, p<0.01 MCF-7-sh3 vs. MCF-7-sh scramble, Figure 4B). Although it was reported that MCF-7 cells have a lower probability to die through caspase-dependent apoptosis [31], we found that 10% of MCF-7-sh scramble cells showed apoptotic morphological characteristics when they were stained with Acridine Orange / Ethidium Bromide, but only about 5% of GPC3 silenced MCF-7 cells exhibited these features (p<0.01 MCF-7-sh3 C2 vs. MCF-7-sh scramble, Figure 4C).

On the other hand, GPC3 overexpression induced a significant reduction in the number of MDA-MB231 viable cells after starving (p<0.001 MDA-MB231-GPC3×1 and MDA-MB231-GPC3×2 vs. MDA-MB231-vector, Figure 4A). Propidium Iodide / Höechst staining revealed that MDA-MB231-GPC3 cells were more susceptible to death under stress conditions (p<0.01 MDA-MB231-GPC3×1 and MDA-MB231-GPC3×2 vs. MDA-MB231-vector, Figure 4B). Although less than 5% of control cells showed morphological evidence of apoptosis, the overexpression of GPC3 induced a high number of MDA-MB231 cells with nuclear and cytoplasmic apoptosis features (p<0.05 MDA-MB231-GPC3×1 and MDA-MB231-GPC3×2 vs. MDA-MB231-vector, Figure 4C).

Similar results were obtained when sublines were submitted to Doxorubicin treatment (data not shown).

##### Migration ability

**Figure 5:**
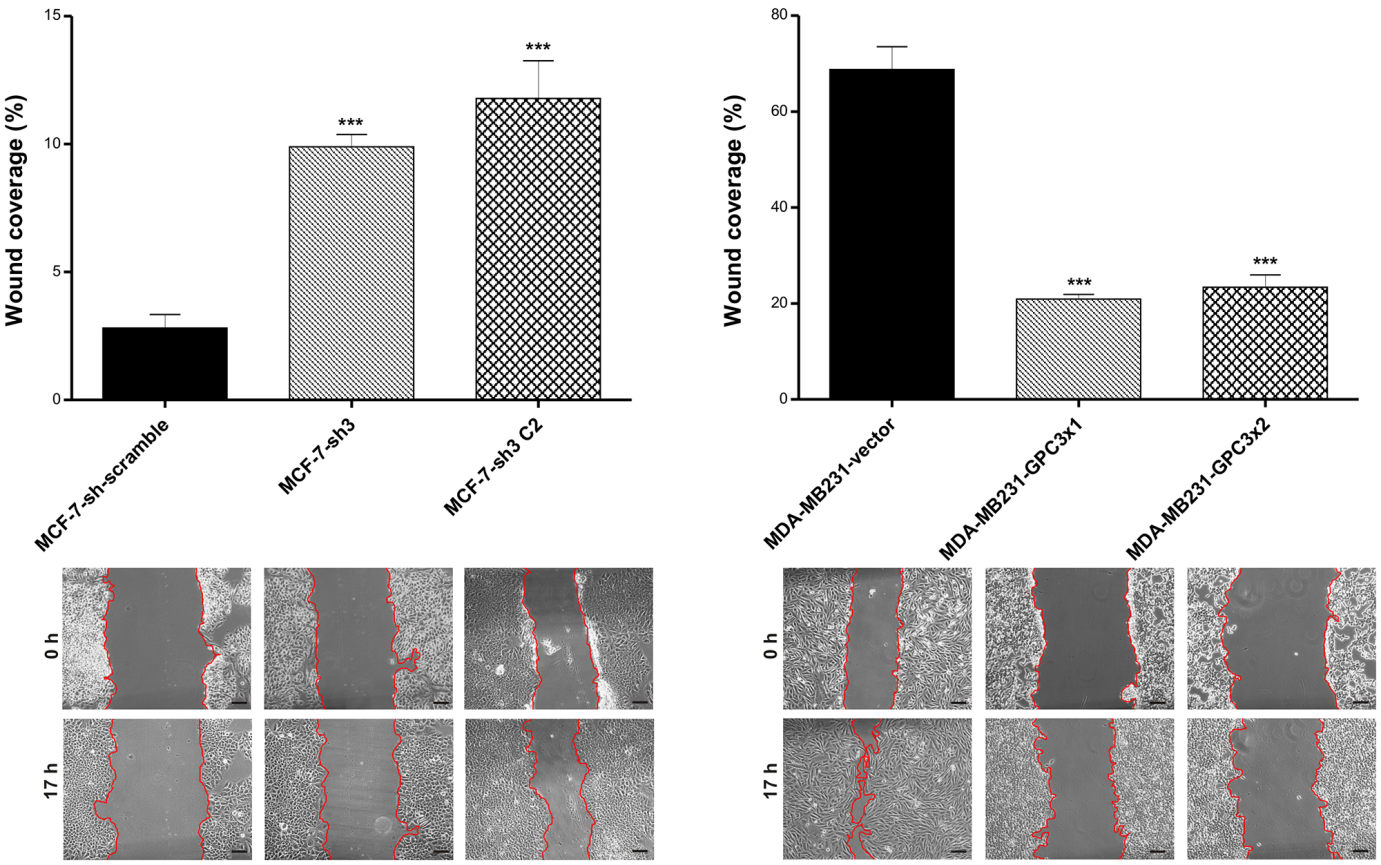
Effect of GPC3 on cell migration Wound healing assays were employed to test the migratory capacity of MCF-7 and MDA-MB231 cell sublines. Migration is expressed as the percentage of wound coverage area (mean ± SD). Representative images are shown (Magnification, x200. Scale bars, 40 μm), where red line indicates the migration front. Data are representative of three independent experiments (***p<0.001 ANOVA, Tuckey test).

*In vitro* wound assays were performed to analyze the migration of engineered cells. Compared to the control group, the silencing of GPC3 stimulated MCF-7 cell migration (p<0.001 MCF-7-sh3 and MCF-7-sh3 C2 vs. MCF-7-sh scramble). On the other hand, we also determined that MDA-MB231-GPC3 cells were significantly less mobile than controls (p<0.001 MDA-MB231-GPC3×1 and MDA-MB231-GPC3×2 vs. MDA-MB231-vector, Figure 5).

#### *In vivo* biological behavior

To confirm the functional role of GPC3 in breast cancer progression, MCF-7 and MDA-MB231 engineered cells were subcutaneously inoculated into nude mice.

We determined that 60% (3/5) of mice inoculated with MCF-7 control as well as MCF-7-sh3 C2 cells developed tumors. On the other hand, GPC3 overexpression significantly decreased MDA-MB231 tumor incidence. In fact, only 20% (2/10) of mice inoculated with MDA-MB231-GPC3×2 cells developed tumors, as opposite to 63.6% (4/6) of mice with tumors in the control group (p< 0.05, Table 3).

**Table 3:** Effect of GPC3 on the *in vivo* cell behavior

	MCF-7-shscramble	MCF-7-sh3C2	MDA-MB231-vector	MDA-MB231-GPC3×2
**Tumorigenicity (%, n/n)**	60 (3/5)	60 (3/5)	63.63 (4/6)	20 (2/10) ^*^
**Tumor Growth Rate (mm^3^/day)**	120 ± 10	130 ± 20	120 ± 30	500 ± 140 ^+++^
**Histological Invasion (%, n/n)**	0 (0/3)	33.33 (1/3)	75 (3/4)	0 (0/2) ^#^
**Spontaneous Metastasis Incidence (%)**	0 (5/5)	60 (3/5) ^&^	50 (3/6)	0 (0/10) ^♠♠^

Tumorigenicity: n of animals that developed tumors/ n of injected animals. Histological invasion: n of animals with tumors presenting local invasion / n of animals with tumors. Spontaneous metastasis incidence: n of animals presenting distant metastasis / n of animals.

+++ p<0.0001 MDA-MB231-GPC3×2 vs. MDA-MB231-vector, Comparing slopes test;

* p<0.05 MDA-MB231-GPC3×2 vs. MDA-MB231-vector,

# p<0.05 MDA-MB231-GPC3×2 vs. MDA-MB231-vector,

& p<0.05 MCF-7-sh3 C2 vs. MCF-7-sh scramble,

♠♠ p<0.01 MDA-MB231-GPC3×2 vs. MDA-MB231-vector, Chi-square test.

**Figure 6:**
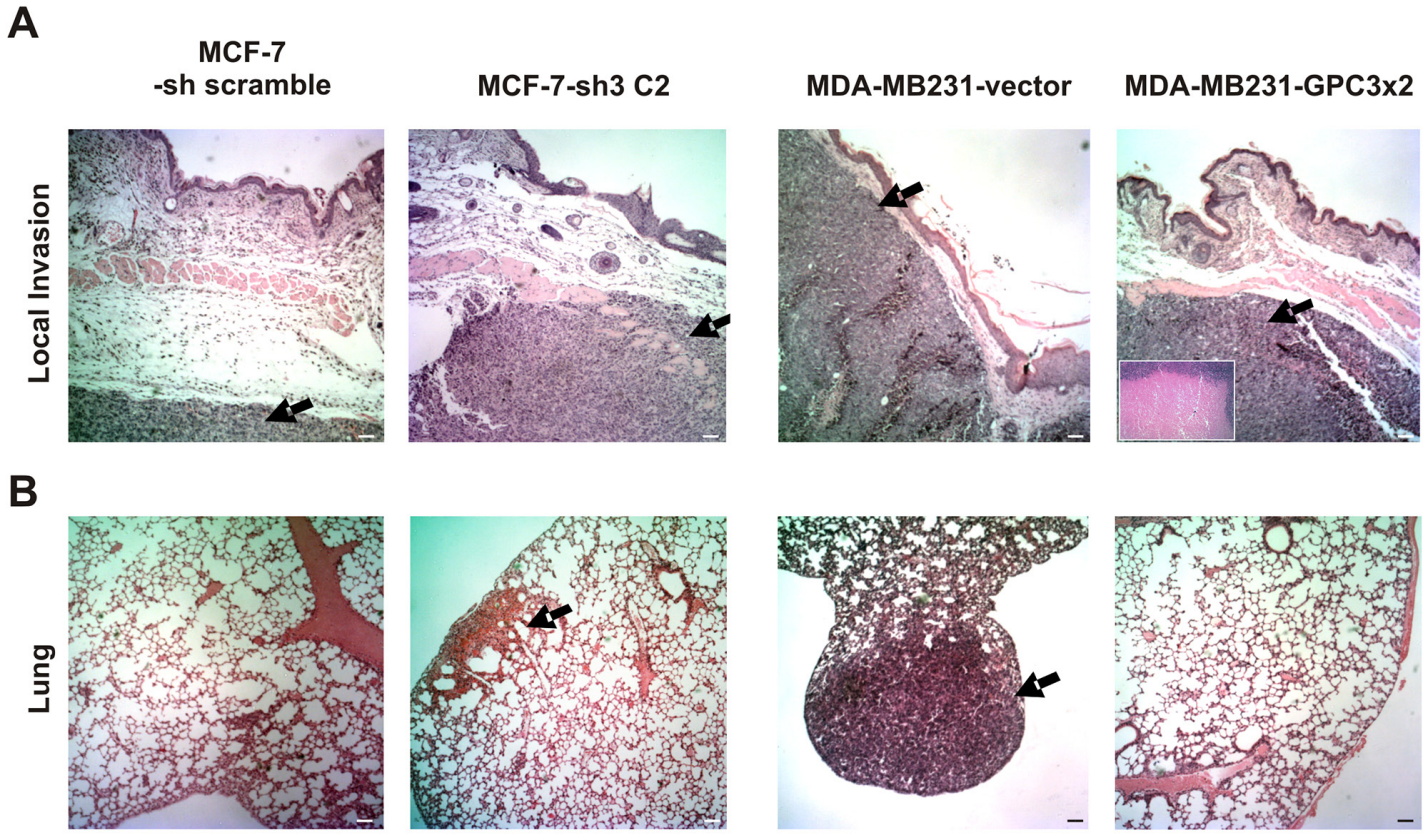
Effect of GPC3 on the *in vivo* cell behavior **A.** Eight-to-ten week old females nude mice (n = 5 to 10 mice/group) were inoculated with MCF-7 (-sh scramble and -sh3 C2) and MDA-MB231 (-vector and -GPC3×2) cells. MCF-7 cells-bearing mice received pellets of 0.5 mg 17-β-estradiol. Representative photographs of tumors are shown (Magnification x40, Scale bar 200 μm). Arrows indicate the tumor. Inset shows MDA-MB231-GPC3×2 tumor core (Magnification x100). **B.** The lungs of tumor-bearing mice were removed and examined for metastases under a dissecting microscope and by histological staining. Representative images are shown (Magnification x40, Scale bar 200 μm). Arrows indicate micrometastatic/metastatic foci. Data are representative of three independent experiments.

All MCF-7 tumors grew at a similar rate. Although few animals inoculated with MDA-MB231-GPC3×2 cells developed tumors, those were bigger than MDA-MB231-vector tumors (p<0.001, Table 3). However, histopathology revealed that GPC3 overexpressing tumors had a large necrotic core, inducing an overestimation of the final tumor volume (Figure 6A, inset).

Histopathological study of s.c. tumors showed differences in the invasiveness behavior. Although 33.33% (1/3) of GPC3 silenced MCF-7 tumors presented an invasion of the s.c. muscle and occasionally the dermis, MCF-7 tumors were unable to invade these tissues (p=0.06 borderline significance, Table 3 and Figure 6A). In turn, GPC3 overexpression inhibited the invasive phenotype of MDA-MB231 cells. We have demonstrated that 75% (3/4) of control tumors invaded the s.c. muscle as well as the dermis, but no invasion was detected in GPC3 overexpressing tumors (p<0.05, Table 3 and Figure 6A).

To evaluate the spontaneous metastatic capacity, lung nodules were studied under lens and by histological staining. All cell sublines only metastasized to lungs, being unable to colonize other organs. No spontaneous surface metastases were found. However, histopathology revealed the presence of parenchymatous nodes and micrometastatic/metastatic foci in the lungs of 60 % (3/5) of mice inoculated with GPC3 silenced MCF-7 cells. It is important to note that the lungs of 100% (5/5) of the animals inoculated with MCF-7-sh control cells were free of metastasis (p<0.05, Table 3 and Figure 6B). At the same time, although lung metastasis was found in 50% (3/6) of animals inoculated with MDA-MB231 control cells, there was no metastasis in lungs of mice injected with MDA-MB231-GPC3 cells (p<0.01, Table 3 and Figure 6B). These results indicate that GPC3 inhibits the metastatic ability of human breast cancer cells.

#### EMT markers

Since we found a severe morphological change in the GPC3 transduced MDA-MB231 cells - from fibroblast-like to squamous-epithelial cell shape - besides modulation on the actin cytoskeleton, growth, death, migration, and *in vivo* behavior, in MCF-7-sh GPC3 and MDA-MB231-GPC3 cells, we decided to study the potential role of GPC3 in the EMT of breast cancer cells. We further examined the expression of the epithelial marker E-Cadherin.

**Figure 7:**
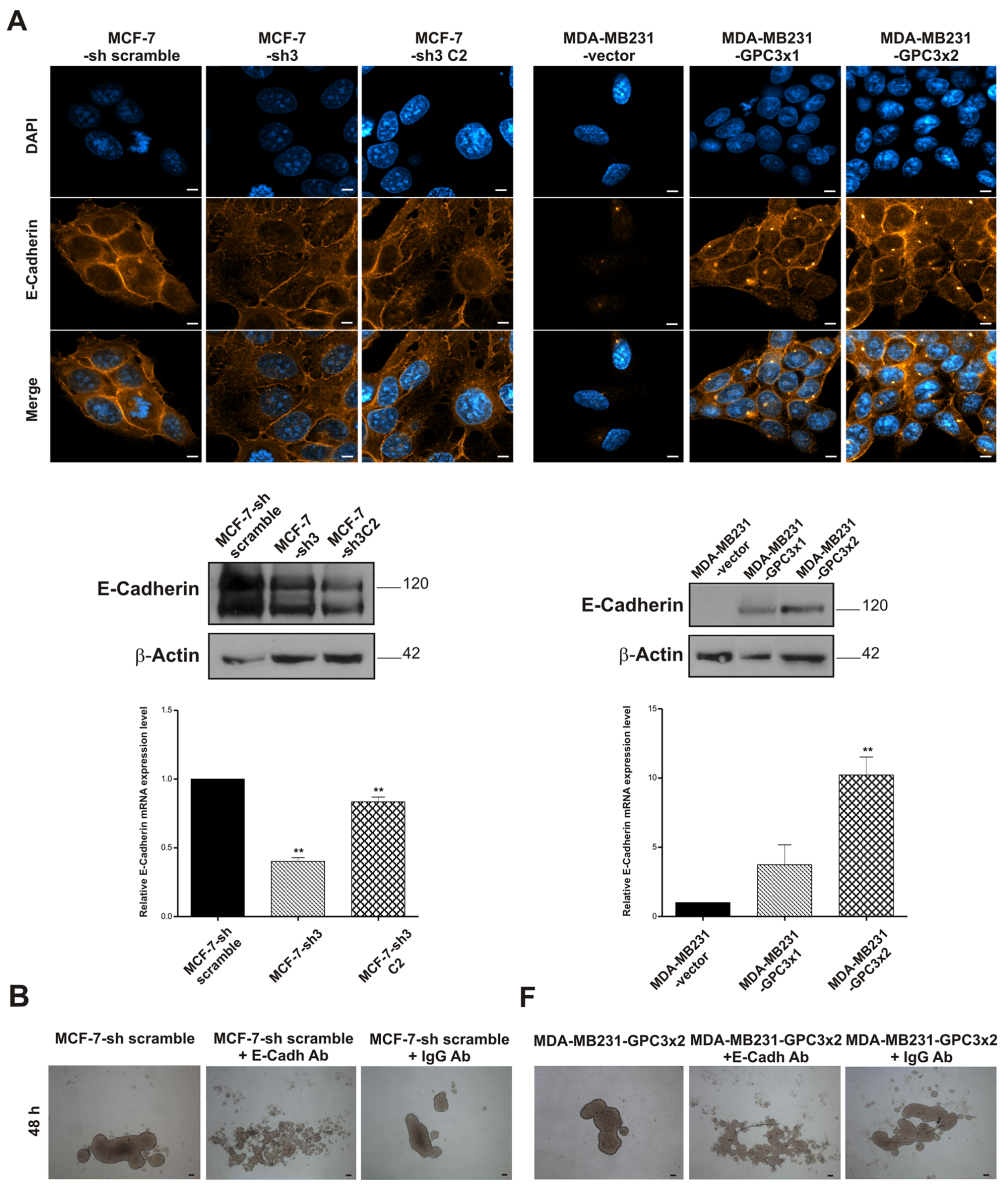
Effect of GPC3 on the expression of the epithelial marker E-Cadherin **A.** E-Cadherin expression was evaluated in MCF-7-sh scramble, MCF-7-sh3 and MCF-7-sh3 C2 as well as MDA-MB231-vector, MDA-MB231-GPC3×1 and MDA-MB231-GPC3×2 by IF (Magnification x1000. Scale bars 8 μm), WB and qRT-PCR. β-Actin was used as a WB internal control, while GAPDH was employed as a qRT-PCR control (**p<0.01 ANOVA, Dunnett test). **B.** The anchorage-independent 3D growth ability of cell sublines, growing in the presence of anti E-Cadherin neutralizing antibody, was examined after 48 h culture. Representative images were taken under inverted phase contrast microscope (Magnification x40. Scale bars 200 μm). Data on the isotype control are shown in the third photo of each panel.

Confocal laser scanning microscopy images of GPC3 silenced MCF-7 cells showed a decrease in the E-Cadherin expression (Figure 7A, upper panel). This was also demonstrated by WB and qRT-PCR (p<0.01 MCF-7-sh3 and MCF-7-sh3 C2 vs. MCF-7-sh scramble, Figure 7A, middle and lower panel). Moreover, although MDA-MB231 cells did not express E-Cadherin, we demonstrated by IF, WB and qRT-PCR that GPC3 induced the re-expression of this epithelial marker (p<0.01 MDA-MB231-GPC3×2 vs. MDA-MB231-vector, Figure 7A). Images of the MDA-MB231-GPC3 cells revealed strong E-Cadherin staining in cell-cell junctions. To confirm the functionality of E-Cadherin, we conducted a homotypic adhesion assay in the presence of anti E-Cadherin neutralizing antibody. As expected, MCF-7 cells failed to form spheroids when E-Cadherin was blocked (Figure 7B). Interestingly, we did not obtain spheroids when MDA-MB231-GPC3 cells were pre-incubated with the E-Cadherin neutralizing antibody (Figure 7B). Assays with an isotype control corroborated the antibody specificity (Figure 7B, third photo of each panel).

**Figure 8:**
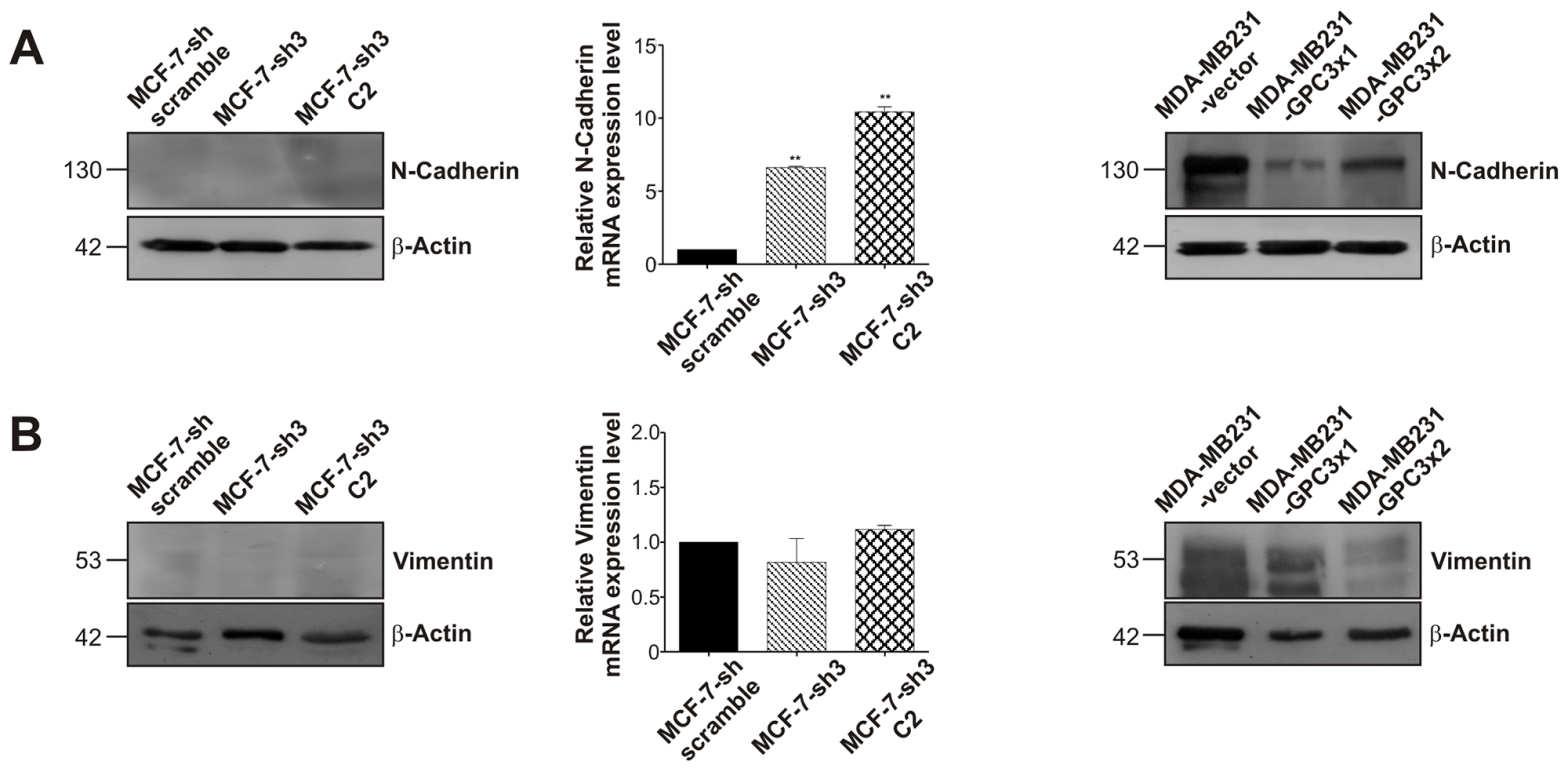
Effect of GPC3 on the expression of the mesenchymal markers N-Cadherin and vimentin **A, B.** WB and qRT-PCR techniques were used to analyze N-Cadherin (A) and vimentin (B) expression. For WB, β-Actin was used as an internal control and numbers on the left indicate molecular mass (kDa). For qRT-PCR, GAPDH was employed as control, and values are expressed as mean ± SD. The data are representative of three independent experiments (**p<0.01 ANOVA, Dunnett test).

Given the radical modulation of the epithelial marker E-Cadherin found in the MDA-MB231 breast cancer sublines, we decided to analyze the expression of the mesenchymal markers N-Cadherin and vimentin. By means of WB, we established that MCF-7 cells were unable to express these EMT-related markers at protein level. However, using qRT-PCR, a significant upregulation of N-Cadherin mRNA levels could be observed when GPC3 was silenced (p<0.01 MCF-7-sh3 and MCF-7-sh3 C2 vs. MCF-7-sh scramble, Figure 8A). Meanwhile, the high expression levels of these mesenchymal markers detected in MDA-MB231 cells were reduced when GPC3 was overexpressed (Figure 8B).

These data demonstrate that GPC3 induced MET in breast cancer cells.

### Regulatory mechanism of E-Cadherin expression: Wnt/β-Catenin signaling pathway and EMT-transcription factors

**Figure 9:**
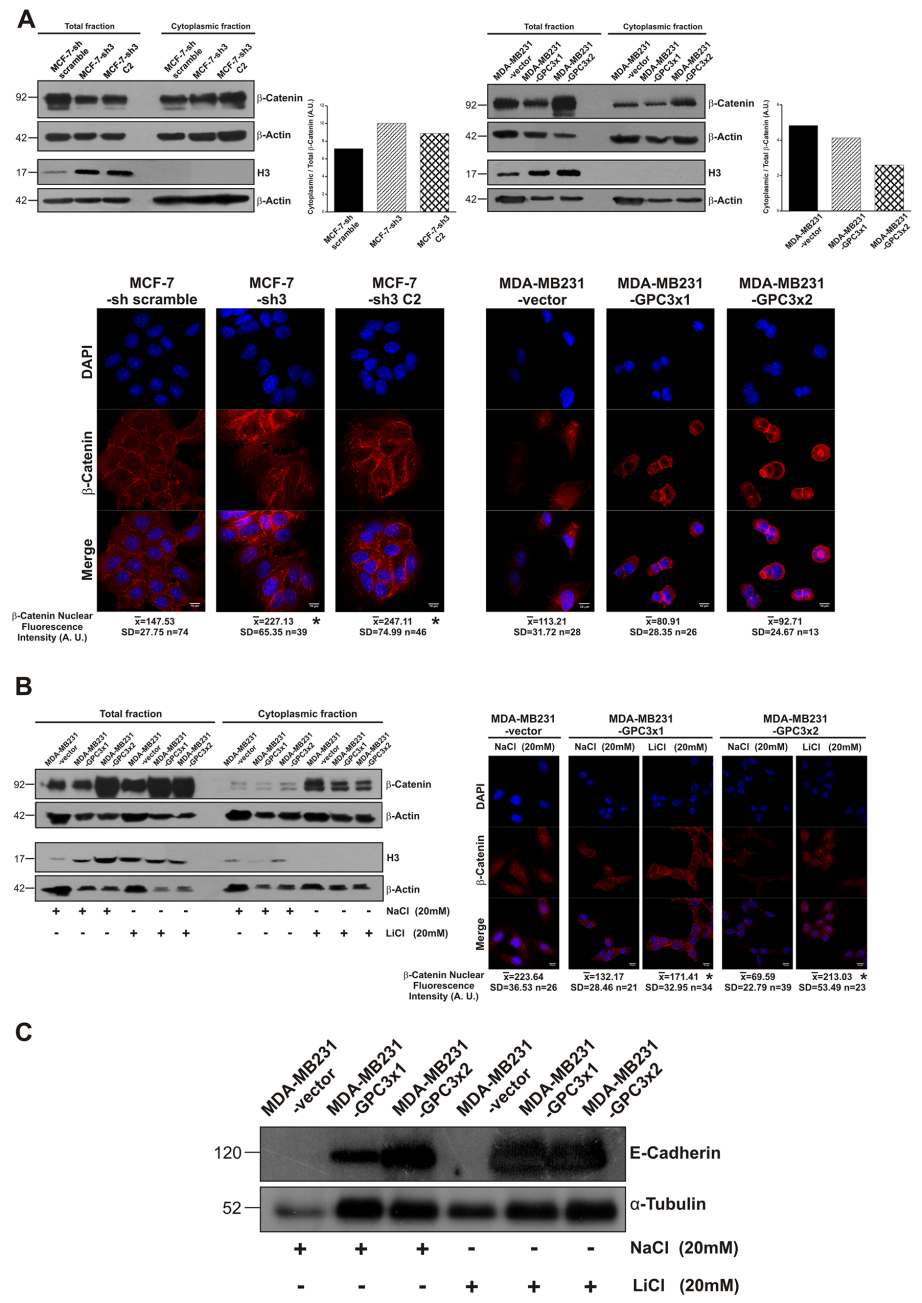
Effect of GPC3 on the canonical Wnt/β-Catenin signaling pathway activity **A.** *Upper panel:* Cytoplasmic and total protein extracts from MCF-7 and MDA-MB231 cell sublines were analyzed by WB using an anti β-Catenin antibody. Loading was standardized by β-Actin levels for each fraction, and the cytoplasmic/total β-Catenin ratio was calculated and presented in the histogram. H3 antibody was employed as nuclear marker. Numbers on the left represent molecular mass (kDa). *Lower panel:* Confocal IF was performed to quantify nuclear β-Catenin intensity, where DAPI was used to define the nucleus. Quantitative microscopy measurements were performed in individual cells (13-76 cells for each subline) and they are indicated below the microphotographies. Representative images are shown (Magnification x600, Scale Bar 10 μm) (*p<0.05 ANOVA, Tuckey test). **B.** MDA-MB231-GPC3 sublines were treated with LiCl (20 mM) or NaCl (20 mM) as control. *Left panel:* The activation of canonical Wnt pathway induced by lithium was tested by cytoplasmic β-Catenin accumulation through WB. *Right panel:* Confocal IF was performed to quantify nuclear β-Catenin intensity (*p<0.05 ANOVA, Tuckey test). C. WB was used to analyze E-Cadherin expression in MDA-MB231-vector, MDA-MB231-GPC3×1 and MDA-MB231-GPC3×2 cells treated with LiCl (20 mM) or NaCl (20 mM) as control. α-Tubulin was used as an internal control and numbers on the left indicate molecular mass (kDa).

It was reported that the canonical Wnt/β-Catenin pathway could participate in the EMT process of breast cancer cells [32]. To reveal the potential mechanism of MET induction by GPC3, we studied the canonical Wnt signaling activation. We analyzed cytoplasmic β-Catenin levels, since it was demonstrated that they are representative of the pathway activity [33]. We used the nuclear marker H3 as a cytoplasmic fraction purity control. WB results showed that cytoplasmic/total β-Catenin ratio increased in GPC3 silenced MCF-7 cells compared to control cells (Figure 9A, upper panel). In addition, MDA-MB231-GPC3 cells displayed lower cytoplasmic/total β-Catenin ratio compared to control cells (Figure 9A, upper panel). To corroborate these results, we decided to analyze nuclear β-Catenin levels as a most direct way to assess canonical Wnt transcriptional activity. Through confocal immunofluorescence we established that the nuclei of GPC3 silenced MCF-7 cells presented about 60% more β-Catenin fluorescence intensity than the nuclei of MCF-7-sh scramble cells (p<0.05 MCF-7-sh3 and MCF-7-sh3 C2 vs. MCF-7-sh scramble). Meanwhile, the nuclear β-Catenin fluorescence level was reduced about 25% in GPC3 overexpressing MDA-MB231 cells (Figure 9A, lower panel). Taken together, both assays indicate that GPC3 is inhibiting canonical Wnt signaling.

Next, we decided to prove whether GPC3 induces MET through the inhibition of Wnt/β-Catenin signaling. To do this, we reversed the inhibitory effect of GPC3 on the canonical pathway in MDA-MB231 cells using the activator LiCl as was described [34]. Lithium acts through inhibition of glycogen synthase kinase-3 beta (GSK-3β), preventing the constitutive proteasome-mediated degradation of cytoplasmic β-Catenin. This results in the accumulation and nuclear translocation of β-Catenin, where it induces β-Catenin/TCF-mediated transcriptional activity [35].

First, we corroborated that LiCl activates the canonical Wnt pathway in our cells. MDA-MB231-vector and MDA-MB231-GPC3 cells were treated with LiCl (or NaCl as control) and analyzed by WB and IF. As seen in Figure 9B, left panel, the treatment with LiCl induced an increase in the cytoplasmic β-Catenin levels, suggesting the activation of canonical Wnt signaling. This was confirmed by IF. Nuclear β-Catenin fluorescence intensity increased 30% in the MDA-MB231-GPC3×1 cells treated with LiCl (p<0.05), whereas this increment was about 200% in MDA-MB231-GPC3×2 lithium treated cells (p<0.05, Figure 9B, right panel).

Later on, we evaluated the expression of E-Cadherin (target downregulated by Wnt pathway). However, no changes were found in E-Cadherin expression when MDA-MB231-GPC3 cells were treated with LiCl (Figure 9C). In other words, GPC3 was able to stimulate the E-Cadherin re-expression in MDA-MB231 cells even when the canonical Wnt signaling is activated. These results indicate that GPC3 induced MET independently of canonical Wnt/β-Catenin signaling.

**Figure 10:**
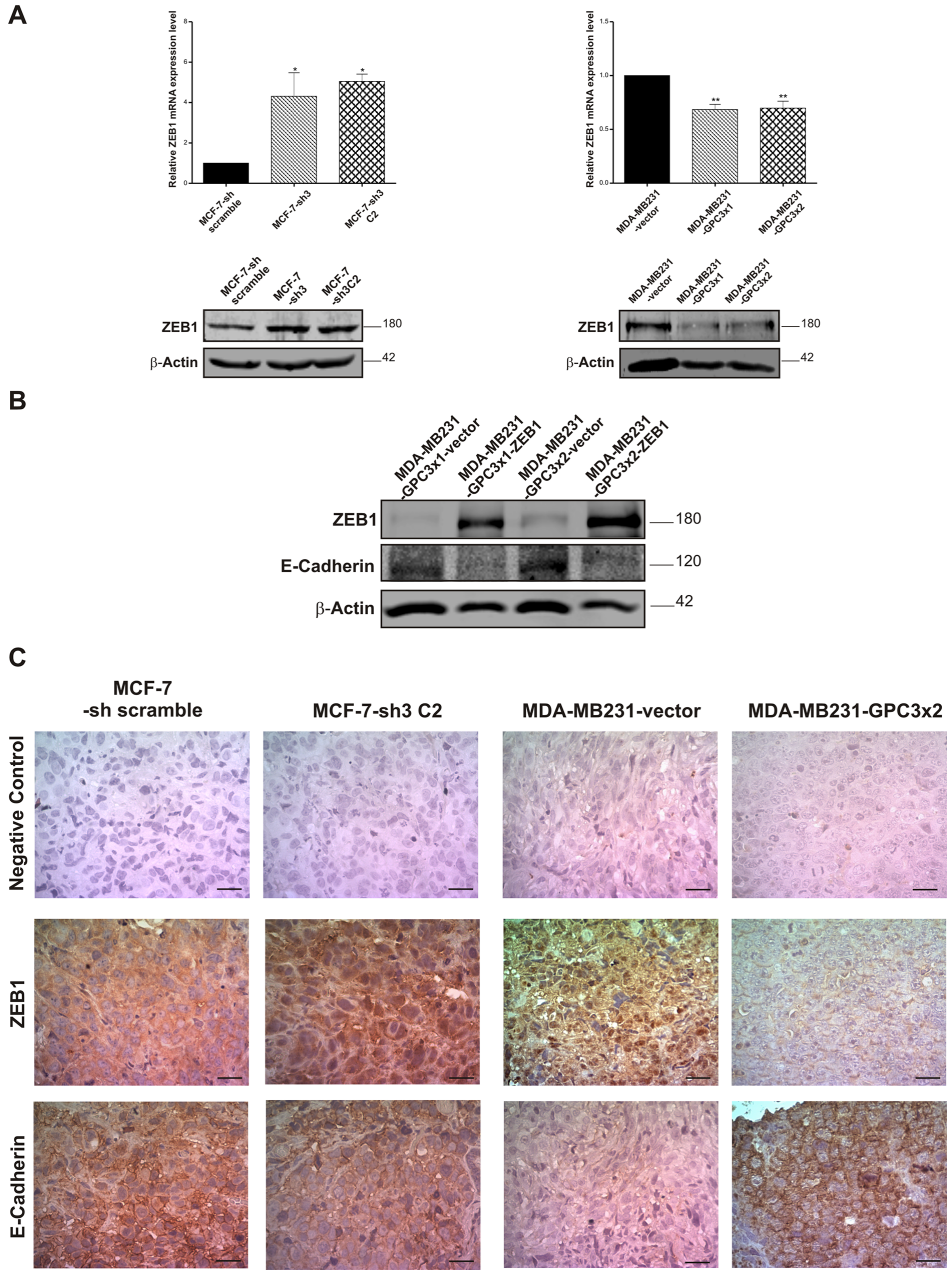
Effect of GPC3 on the expression of the transcriptional repressor ZEB1 **A.** ZEB1 mRNA levels were analyzed by qRT-PCR (*upper panel*) and ZEB1 protein expression was determined by WB (*lower panel*), in MCF-7 and MDA-MB231cell sublines. In qRT-PCR reactions, GAPDH was used as an internal control. Values are expressed as mean ± SD (*p<0.05, **p<0.01 ANOVA, Dunnett test). In WB, loading was standardized by β-Actin levels. Numbers on the right represent molecular mass (kDa). The data are representative of two independent experiments. **B.** Protein extracts from MDA-MB231-GPC3 cells transfected with a plasmid encoding ZEB1 (or with the empty vector as control) were analyzed by WB. The expression levels of ZEB1 and E-Cadherin were determined. β-Actin was used as an internal control. Numbers on the right indicate molecular mass (kDa). The data are representative of three independent experiments. **C.** ZEB1 and E-Cadherin expression was evaluated at protein level by IHC, in MCF-7-sh scramble, MCF-7-sh3 C2, MDA-MB231-vector and MDA-MB231-GPC3×2 tumors. Representative images are shown. Original magnification x400, scale bar 20 μm.

It has been reported that EMT-transcription factors can regulate E-Cadherin expression in specific cellular contexts and control the EMT program [5]. Therefore, the mRNA levels of SNAIL1, SNAIL2 (SLUG), and ZEB1 were determined by qRT-PCR. The results showed that ZEB1 expression was significantly increased in GPC3 silenced MCF-7 cells (p<0.05 MCF-7-sh3 and MCF-7-sh3 C2 vs. MCF-7-sh scramble, Figure 10A, upper panel). Conversely, MDA-MB231 cells significantly decreased the ZEB1 mRNA levels when GPC3 was overexpressed (p<0.01 MDA-MB231-GPC3×1 and MDA-MB231-GPC3×2 vs. MDA-MB231-vector, Figure 10A, upper panel). This modulation was confirmed at protein level by WB (Figure 10A, lower panel). The other transcription repressors did not present relevant changes (data not shown).

In order to corroborate whether GPC3 induced the re-expression of E-Cadherin in MDA-MB231 cells through the downregulation of the transcriptional repressor ZEB1, we transfected MDA-MB231-GPC3 cells with a vector encoding ZEB1 (empty vector was used as control). As seen in Figure 10B, the expression of E-Cadherin was inhibited when MDA-MB231-GPC3 cells overexpressed ZEB1. In other words, GPC3 induces the E-Cadherin upregulation by inhibiting ZEB1 expression.

Next, we examined whether the *in vitro* GPC3 effect on the expression of ZEB1 and E-Cadherin was maintained *in vivo*. With this aim, MCF-7-sh scramble, MCF-7-sh3 C2, MDA-MB231-vector and MDA-MB231-GPC3×2 tumors were analyzed by IHC. The assays revealed ZEB1 expression in nuclei, cytoplasm and atypically in the stroma of different tumors (Figure 10C). For analysis, only unequivocal nuclear staining was accepted as positive [36, 37]. Nuclear ZEB1 was detected in 61.5 ± 13.8% of cells from MCF-7-sh scramble tumors, compared to 97.2 ± 8.0% of positive cells found in MCF-7-sh3 C2 tumors (p<0.001). Moreover, 73.0 ± 15.1% of cells from MDA-MB231-vector tumors showed positive staining for ZEB1, while 33.2 ± 3.2% of cells of GPC3 overexpressing tumors did it (p<0.01). On the other hand, IHC revealed positive E-Cadherin staining mainly at the plasma membrane of the tumor cells, as expected. As shown in Figure 10C, all cells from MCF-7-sh scramble tumors were positive for this epithelial marker. Although the most of the cells of the MCF-7-sh3 C2 tumors remained positive for E-Cadherin, we observed a decrease in the intensity of staining. Contrarily, most of MDA-MB231-vector tumor cells were negative for E-Cadherin, while 95.0 ± 5.2% of MDA-MB231-GPC3 tumor cells expressed this protein (p<0.001).

## DISCUSSION

The plasticity that cells show during the EMT process is crucial to tumor metastasis development [3]. Cancer cells undergoing EMT can acquire invasion capability and enter surrounding tissues, a critical step of the metastatic cascade. Moreover, the effect of EMT not only includes increasing migration, invasion and metastatic potential, but also the acquisition of chemoresistance [3]. So, we consider that discovering molecules able to revert EMT (or to promote MET) is key for the development of effective anticancer therapeutics.

Although historically only mechanical and structural functions were attributed to proteoglycans, more recent studies have highlighted their contribution in cell behavior control. In this study, we emphasize the inhibitory role of GPC3 on breast cancer progression. In addition, our findings underline the intimate connections between GPC3 and the EMT program operating in tumorigenesis.

Here we study the role of GPC3 in breast cancer biology. GPC3 silencing induced the reorganization of the actin cytoskeleton, with the acquisition of few stress fibers by MCF-7 cells. Most notably, GPC3 overexpression in MDA-MB231 cells stimulated a dramatic morphological change, from a mesenchymal to an epithelial phenotype. Even more, the loss of stress fibers supports the idea that GPC3 induces a transition to an epithelial phenotype.

We also showed that GPC3 expression decreased the clonogenic efficiency of breast cancer cells, since GPC3 silenced MCF-7 cells showed higher aptitude to grow at low density while GPC3 overexpressing MDA-MB231 cells were less clonogenic than their controls. Supporting these results, it was reported that overexpression of GPC3 in renal carcinoma [23] and in ovarian cancer [24, 25] cell lines, reduced their clonogenic efficiency. Our results also indicated that silencing GPC3 did not induce significant changes in the ability of MCF-7 cells to grow in 3D spatial organization, but its overexpression stimulated the formation of large MDA-MB231 spheroids. This is, to our knowledge, the first report indicating that GPC3 is able to modulate anchorage-independent 3D growth in breast cancer cells. Simultaneously with our work, Gao et. al. have reported that blocking the GPC3 heparan sulphate chains inhibited *in vitro* hepatocarcinoma spheroid formation [38]. All together, our results suggest that GPC3 is able to modulate different growth properties of breast cancer cells.

On the other hand, we determined that GPC3 expression increased the susceptibility to death. Although it was informed that the overexpression of GPC3 did not affect the apoptosis of renal carcinoma cells [23], our studies showed that MCF-7-sh GPC3 cells were less susceptible to death, while MDA-MB231-GPC3 died more after starvation. This behavior was also observed when cells were treated with Doxorubicin. In association, we have previously demonstrated that the re-expression of GPC3 induced a decrease in the apoptosis resistance acquired by the LM3 murine mammary adenocarcinoma cells [28, 30]. This pro-apoptotic role is supported by a previous report revealing that MCF-7 cells transfected with GPC3 gene generated fewer clones that those cells transfected with an inactive mutant of GPC3 [39]. On the contrary, it was shown that the GPC3 silencing leads to an increase in apoptosis of hepatocarcinoma cells [40]. In summary, our results show that GPC3 would act as an inductor of cell death in stressed breast cancer cells. The reported discrepancies reinforce the idea of the GPC3 tissue specific role, highlighting once more the importance of tumor microenvironment.

Our wound healing assays indicated that silencing GPC3 stimulated motility of MCF-7 cells, while GPC3 overexpression blocked MDA-MB231 cell migration. These results suggest that GPC3 inhibits the migratory ability of breast cancer cells. The same inhibitory effect has been reported for murine mammary tumor cells as well as for human ovarian cancer cell lines [28, 29, 41]. In contrast, GPC3 silencing leads to a decrease in the migratory capacity of hepatocellular carcinoma cell lines [42, 43].

To analyze whether GPC3 is able to modulate the invasive and metastatic behavior of the human breast cancer cell lines, we performed *in vivo* assays. We showed that GPC3 expressing s.c. tumors are less invasive and metastatic. Although MCF-7 control tumors grew *in situ*, GPC3 silencing stimulated these tumors to invade adjacent tissues and to metastasize. MDA-MB231 control tumors invaded the muscle and dermis as well as developed metastasis, but GPC3 overexpression inhibited these capabilities. In the same way, we have previously reported that GPC3 re-expression reduced the ability of the LM3 murine mammary adenocarcinoma cells to invade the dermis and to form lung metastasis [28]. Therefore, we have suggested that GPC3 acts as a metastasis suppressor in breast cancer [3, 28]. In association, a paper recently published identifies GPC3 as a potential metastasis suppressor in gastric cancer [26].

All together, our *in vitro* and *in vivo* results show that GPC3 is able to promote MET in mammary tumor cells, inducing phenotypic changes and regulating growth, death, migration and invasive/metastatic ability. To confirm this hypothesis, we evaluated the expression of mesenchymal and epithelial markers. We found that the downregulation of GPC3 in MCF-7 cells inhibited E-Cadherin expression and increased N-Cadherin mRNA levels. Most notable was the difference found in the MDA-MB231 cells, since GPC3 forced the re-expression of the epithelial marker E-Cadherin, while the mesenchymal markers N-Cadherin and vimentin were downregulated. Interestingly, we demonstrated that the E-Cadherin re-expressed by MDA-MB231-GPC3 cells was functional, since these cells acquired the ability to form E-Cadherin-dependent spheroids. Since E-Cadherin suppression in cancer cells enhances the development of migratory and invasive phenotype and facilitates dissociation from the surrounding extracellular matrix of the primary tumor site [5], we suggest that GPC3 would induce MET by regulating E-Cadherin expression.

There are a number of signaling pathways involved in the EMT process. Among them, the classical Wnt/β-Catenin pathway could participate in the EMT progression of cancer cells [29, 44, 45]. We demonstrated that cytoplasmic/total β-Catenin ratio increased in GPC3 silenced MCF-7 cells, while this ratio decreased in GPC3 overexpressing MDA-MB231 sublines. This was also demonstrated by IF, where the levels of nuclear β-Catenin were analyzed. The experiments performed during our study confirm that the activity of the classical Wnt/β-Catenin pathway is inhibited by GPC3. This result is in accordance with our previous work, where we demonstrated that GPC3 is a modulator of Wnt signaling in murine mammary cancer cells, by inhibiting the canonical pathway and activating the non-canonical one [29]. Once again, highlighting the opposite role of GPC3 in hepatocarcinoma cells, it was informed that this glypican is able to activate Wnt/β-Catenin signaling pathway in this pathology [40].

Here we showed that the role of GPC3 in the EMT process is not related to the canonical Wnt signaling. We evaluated the E-Cadherin expression levels when the Wnt/β-Catenin pathway inhibition induced by GPC3 was reverted employing the activator LiCl. We determined that when MDA-MB231-GPC3 cells were incubated with LiCl, the expression levels of E-Cadherin did not change. Therefore, these results show that GPC3 is capable to induce MET independently of the canonical Wnt signaling pathway.

Although Wnt signaling is important for the regulation of the EMT program in diverse tumor cells, the above results are not unexpected. When canonical Wnt pathway is activated, β-Catenin translocates to the nucleus and forms a complex with T-cell factor/lymphoid enhancer factor (TCF/LEF) initiating the transcription of Wnt target genes, including SNAIL1 [46]. SNAIL1 is one of the EMT-inducing transcription factors able to repress E-Cadherin. However, SNAIL1 expression was not modulated in our cells. There are others transcription factors, such as SNAIL2 (SLUG), ZEB1 and ZEB2, that directly or indirectly repress the hallmark of epithelial phenotype, the E-Cadherin expression [8]. Our results showed that GPC3 silencing induced the upregulation of ZEB1 in MCF-7 cells, while MDA-MB231-GPC3 cells presented lower levels of ZEB1 as compared to controls. To confirm that GPC3 modulates the E-Cadherin expression downregulating ZEB1, MDA-MB231-GPC3 cells were transfected with a vector encoding ZEB1. We determined that GPC3 is unable to induce the E-Cadherin re-expression in MDA-MB231 cells if ZEB1 is overexpressed. We also confirmed by IHC, that the modulation of ZEB1/E-Cadherin induced by GPC3 is maintained in *in vivo* tumors. All together, our results demonstrate that GPC3 induces the E-Cadherin upregulation through ZEB1 modulation. In association, Qin and collaborators have recently reported that hTERT promotes the colorectal cancer cells EMT independent of Wnt, through the ZEB1 pathway [47]. It has been reported that TGF-β, a major inducer of EMT [48], regulates the expression of target genes like ZEB [49]. Therefore, TGF-β pathway would be an alternative mechanism to mediate the ZEB1 modulation induced by GPC3. However, details of this EMT regulatory network remain unclear. Future mechanistic studies are needed to test this hypothesis.

In summary, our results indicate that GPC3 can modulate several mechanisms involved in mammary tumorigenesis and malignant progression. GPC3 induces MET through ZEB1 pathway, and controls growth, death, migration and metastatic spread of breast cancer cells. GPC3 might be a new therapeutic target for preventing breast cancer cell metastasis.

## MATERIALS AND METHODS

### Tumor cell lines

The human breast cancer cell lines MDA-MB231, Hs578T, ZR-75-1 and MCF-7 (Table 1) were obtained from the ATCC in 2008 (Manassas, VA, USA). The authentication of the cell lines used in this work were performed by examining up to 22 polymorphic loci for human and cell line STR profiling in accordance with the standard ASN-0002-2011 (DDC Medical, Fairfield, OH, USA. Validation date: July 16^th^ 2015). An electropherogram provided the data generated by the STR DNA analysis.

All cell lines were grown in RPMI 1640 medium (Gibco Life Technologies, Carlsbad, CA, USA) with non-essential aminoacids and 2 mM L-glutamine, supplemented with 10% fetal calf serum (FCS) (Internegocios, BA, Argentina) and 80 mg/ml gentamicin, at 37°C in a humidified 5% CO_2_ - air atmosphere.

### Quantitative Real-Time PCR (qRT-PCR)

Total RNA was extracted using TRIZOL reagent (Invitrogen Life Technologies, Carlsbad, CA, USA) according to the manufacturer's instructions. RNA was quantified in a Nanodrop (Thermo 2000 spectrophotometer) and cDNA was synthesized from 1 μg of RNA previously treated with 10 Units of DNAsa I (Invitrogen Life Technologies, Carlsbad, CA, USA), using iScript cDNA synthesis kit (Bio-Rad Life Science, Hercules, CA, USA). The reaction conditions were as follows: a period of 5 min at 25°C, 30 min at 55°C and 5 min at 95°C. The resulting cDNA was subsequently treated with 1 Unit of RNase H (GE Healthcare, Little Chalfont, Buckinghamshire, UK).

qRT-PCR reactions contained: 3 μl cDNA (1:10), 6 μl 2X SYBR Green Master Mix (Applied Biosystems Life Technologies, Carlsbad, CA, USA), and forward and reverse primers for GPC3, E-Cadherin, N-Cadherin, vimentin, SNAIL1, SNAIL2 (SLUG), ZEB1 and GAPDH (Table 2). Reactions were completed in a termocyler/detector REAL TIME C1000 CFX96 (Biorad, Hercules, CA, USA). Cycle conditions were: 50°C 2 min, 95°C 10 min and 40 cycles at 95°C for 15 sec and 60°C during 1 min. 2−^ΔΔCt^ was used to calculate relative gene/GAPDH expression. All samples were run in triplicate.

### Western blot (WB)

Confluent monolayers were washed three times with ice cold PBS and then lysed with Lysis Buffer (PBS-1% Triton X-100) containing protease-inhibitors (Sigma-Aldrich, Saint Luis, MO, USA). In order to analyze whether canonical Wnt signaling is involved in E-Cadherin regulation, cells were treated 2 h with 20 mM LiCl (activator of canonical pathway) or with 20 mM NaCl as control. For analysis of cytoplasmic β-Catenin levels, cytoplasmic extracts were obtained using a saponin buffer, as previously described [29]. Briefly, cells were lysed with 250 ml of saponin lysis buffer (25 mM Hepes, 75 mM potassium acetate, 0.1% saponin, phosphatase inhibitor cocktail and protease inhibitors). The extraction procedure was carried out twice; the extracts were pooled and then centrifuged.

Protein content of cell samples was determined by Bradford method. The samples were boiled in Laemmli sample buffer with 5% β-mercaptoethanol. WB analyses were carried out using a sodium dodecyl sulfate-polyacrylamide gel electrophoresis (SDS-PAGE). Electrophoresis gels were transferred to PVDF membranes using “Mini Trans-Blot module” (BioRad, Hercules, CA, USA). Non-specific binding was blocked by incubation of the membrane with TBS 5% skim milk for an hour. Then, membranes were incubated with the specific antibodies overnight at 4°C (1:1500 for hGPC3 mouse monoclonal antibody 1G12, kindly provided by Dr. Filmus; 1:1000 for E-Cadherin mouse monoclonal antibody, BD Bioscience, San Jose, CA, USA; 1:1000 for β-Catenin mouse monoclonal antibody, BD Bioscience, San Jose, CA, USA; 1:1000 for vimentin rabbit monoclonal Abcam, Cambridge, MA, USA; 1:1000 for N-Cadherin rabbit polyclonal antibody, Abcam, Cambridge, MA, USA; 1:500 for ZEB1 H102 rabbit polyclonal antibody, Santa Cruz Biotechnology, Dallas, TX, USA; 1:15000 for H3 rabbit polyclonal antibody, EMD Millipore, Billerica, MA, USA; 1:5000 for β-Actin mouse monoclonal antibody Santa Cruz Biotechnology, Dallas, TX, USA; 1:1000 for α-Tubulin rabbit monoclonal antibody, Cell Signaling, Danvers, MA, USA). Membranes were subsequently blotted with peroxidase-conjugated goat anti-mouse (1:5000, Santa Cruz Biotechnology, Dallas TX, USA) or goat anti-rabbit (1:5000, Sigma-Aldrich St. Louis, MO, USA) secondary antibodies, for 1 h at room temperature. After that, detections were performed using ECL western blot reagents (Amersham-GE Healthcare, Little Chalfont, Buckinghamshire, UK). Electrophoretic band images were obtained and analyzed by densitometry (Optical Density, OD) by ImageJ1.49m program. In all WB, OD of each protein was standardized to the corresponding loading control OD (β-Actin or α-Tubulin as corresponded). To analyze cytoplasmic/total β-Catenin ratio, total and cytoplasmic extracts were resolved into the same gel. The cytoplasmic β-Catenin/β-Actin and total β-Catenin/β-Actin relation values were calculated, and the ratio between these values (cytoplasmic/total) represented cytoplasmic β-Catenin accumulation.

### Immunofluorescence (IF)

Cells were grown on glass coverslips, washed twice with PBS, fixed in 4% formaldehyde/PBS at RT for 15 min, permeabilized with PBS-0.1% Triton X-100 for 10 min at 37°C and then blocked with PBS 5% BSA (ChemCruz, Dallas, TX, USA) for 1 h. E-Cadherin was detected by incubation with a primary monoclonal antibody (BD Bioscience, San Jose, CA, USA) diluted in PBS+5% BSA (1:100), followed by incubation with an anti-mouse IgG-Alexa 546 secondary antibody (1:500) for 1 h (Invitrogen-Thermo Fisher Scientific, Waltham, MA, USA). Nuclei were stained with DAPI. Images were obtained in an Olympus Fluo view FV 1000 microscope.

For F-Actin staining, coverslips were incubated for 45 min at RT with phalloidin-FITC (Sigma-Aldrich, Saint Luis, MO, USA) (1:400) and cell nuclei were counter-stained with DAPI. The coverslips were mounted with Mowiol 4-88 (Calbiochem, Darmstadt, Germany). Cells were imaged by confocal laser scanning microscopy, which was performed with an Olympus Fluo view FV 1000 microscope, using an Olympus 60×/1.20 NA UPLAN APO water immersion objective and a 3x digital zoom. Excitation and emission filters were given as follows: DAPI: excitation, 405 nm; emission, band pass: 430 to 470 nm. FITC: excitation, 488 nm; emission, band pass 505 to 525 nm. Confocal images were processed for presentation with FIJI (https://fiji.sc). Background of each channel was subtracted. Briefly, FIJI software was used to generate line profiles (13 μm length for MCF-7 cells, 20 μm length for MDA-MB231 control cells and 10 μm length for MDA-MB231-GPC3×1 and -GPC3×2 cells). A graphic depiction was then generated where the x-axis represented the distance across the cell and the y-axis represented the level of fluorescence. We randomly selected 12 cells of each group for graphic depicting.

For nuclear β-Catenin analysis, monolayers were treated, as appropriate, with 20 mM LiCl or 20 mM NaCl as control for 2 h. Coverslips were fixed in 70% ethanol at −20°C for 10 min, permeabilized with PBS-0.1% Triton X-100 for 10 min at 37°C and blocked with PBS 3% BSA (ChemCruz, Dallas, TX, USA) for 1 h. β-Catenin antibody (BD Bioscience, San Jose, CA, USA) diluted 1:600 in 3% BSA was incubated ON at 4°C, followed by incubation with an anti-mouse IgG-Alexa 546 secondary antibody (1:2000) for 1 h at RT (Invitrogen-Thermo Fisher Scientific, Waltham, MA, USA). Cell nuclei were counter-stained with DAPI. The coverslips were mounted with Mowiol 4-88 (Calbiochem, Darmstadt, Germany). Olympus Fluoview FV 1000 confocal microscope with a UPLSAPO 60× 1.2 NA water immersion objective and a 2x digital zoom was employed. Excitation and emission filters were as follows: excitation DAPI, 405 nm; emission DAPI, BP: 430–470 nm; excitation Alexa fluor 555, 543 nm; emission Alexa fluor 555, BP: 560– 620 nm. We always used the sequential mode for image acquisition. All the quantitative microscopy measurements were performed in individual cells (13-76 cells for each treatment or condition). Confocal microscope images were processed with FIJI (https://fiji.sc). Channel backgrounds (mean of empty region) were subtracted. Segmentation of the nuclear compartment was performed for each cell using the DAPI signal. With this mask the nuclear β-Catenin signal was defined as the product of the DAPI mask and total β-Catenin signal. To calculate the nuclear fluorescence intensity for each cell, automatic recognition of the nuclei was performed to the previous segmented image using the Analyze Particles plug-in of FIJI. We evaluated the correct recognition of each nucleus visually.

### Immunohistochemistry (IHC)

Formalin-fixed, paraffin-embedded MCF-7-sh scramble, MCF-7-sh3 C2, MDA-MB231-vector and MDA-MB231-GPC3×2 tumors were cut at a thickness of 5 mm. Dewaxed sections in 0.01 M citrate buffer (pH 6.0) were heated at 90°C for 6 min. Endogenous peroxidase activity was blocked with 0.3% hydrogen peroxide in distilled water for 30 min, and non-specific immunoglobulin binding was blocked by incubation with 10% normal serum, during 60 min. Sections were incubated ON at 4°C with the primary antibody for E-Cadherin (1:50, BD Bioscience, San Jose, CA, USA) or ZEB1 (1:50, Santa Cruz Biotechnology, Dallas, TX, USA). Sections were rinsed and incubated for 30 min with universal secondary antibodies (1:15, Universal kit, Vector Laboratories, Burlingame-CA-USA). Slides were revealed by employing the Vectastain ABC Universal kit (Vector Laboratories, Burlingame-CA-USA) and the 3,3′-diaminobenzidine chromogen (7%) plus 3% H_2_O_2_ in PBS and counterstained with Meyer's hematoxylin.

All series included negative controls where the primary antibody was omitted. For analysis, images of multiple fields were captured. These images were then exported to FIJI (https://fiji.sc), the background subtracted and the Color De convolution plug-in applied according to the hematoxylin/DAB setting (H DAB) to provide two separate images representing the counter-staining and DAB immunostaining [50]. This DAB-specific image was then standardized to threshold, and the percentage of area occupied by the identified positive staining calculated for tumor using standard algorithms in FIJI. T student test (2-tailed) was used to evaluate the proportion expressing ZEB1 or E-Cadherin protein in MCF-7-sh scramble vs. –sh3 C2 and MDA-MB231-vector vs. –GPC3×2 tumors.

### Silencing of GPC3 gene in MCF-7 cell line

To inhibit GPC3 expression, MCF-7 cells were transfected employing Fugene (Roche, Indianapolis, IN, USA), with the commercial plasmid pGPHI/GFP/Neo coding three shRNA-GPC3 different sequences (named sh1, sh2 and sh3), one shRNA-scramble (negative control) and one shRNA-GAPDH (positive control) (GenePharma Co., Shanghai, China) (Table 4). The selection was carried out with 400 μg/ml of G418 (Gibco LifeTechnologies, Carlsbad, CA, USA). We cloned the MC-F-7 transfected cells by limiting dilution. We selected MCF-7-sh3 GPC3 bulk (named MCF7-sh3), MCF-7-sh3 GPC3 clone 2 (named MCF-7-sh3 C2) and MCF-7-sh scramble bulk (named MCF-7-sh-scramble) sublines for studies.

**Table 4:** Sequences of primers used to qRT-PCR reactions

Name	Target sequence
*GPC3*	F: 5′-GACGCCACCTGTCACCAAGT-3′ R: 5′-AAACTCCCGTGCCAGGATC-3′
*E-Cadherin*	F: 5′-GGTGCTCTTCCAGGAACCTC-3′ R: 5′-GGAAACTCTCTCGGTCCAGC-3′
*N-Cadherin*	F: 5′-GTACAGTGTAACTGGGCCAGG-3′ R: 5′-GATCCAAGTCCAGCTGCCACTG-3′
*Vimentin*	F: 5′-CCAAACTTTTCCTCCCTGAACC-3′ R: 5′-GTGATGCTGAGAAGTTTCGTTGA-3′
*SNAIL2 (SLUG)*	F: 5′-TCGGACCCACACATTACCTTG-3′ R: 5′-TTCTCCCCCGTGTGAGTTCTA-3′
*SNAIL1*	F: 5′-CCAGTGCCTCGACCACTATG-3′ R: 5′-CTGCTGGAAGGTAAACTCTGGA-3′
*ZEB1*	F: 5′-TGCACTGAGTGTGGAAAAAGC-3′ R: 5′-TTGCAGTTTGGGCATTCATA-3′
*GAPDH*	F: 5′-ACCCACTCCTCCACCTTTGA-3′ R: 5′-ACGAATTTGGCTACAGCAACAG-3′

### Overexpression of GPC3 gene in MDA-MB231 cell line

To overexpress GPC3, MDA-MB231 cells were infected with a lentivirus containing the pLV-GPC3-GFP or pLV-GFP (negative control) vector. Virus packaging was performed in 293-T cells after co-transfection of the target plasmid using Fugene (Roche, Indianapolis, IN, USA). Condition media was used for virus titration (pLV-GFP: 1.8 × 10^7^ cfu/ml, pLV-GPC3-GFP: 2 × 10^6^ cfu/ml) 48 h post-transfection. MDA-MB231 cells (1 × 10^5^) were infected, once or twice, with the filtered lentivirus and 2 mg/ml polybrene (Sigma-Aldrich, Saint Luis, MO, USA). Transduced cells were grown for 15 days with complete medium plus 10% FCS containing 400 μg/ml of G418 (Gibco LifeTechnologies, Carlsbad, CA, USA). The selected colonies were isolated and expanded.

We obtained the following sublines: MDA-MB231-GPC3×1 (infected once with GPC3), MDA-MB231-GPC3×2 (infected twice with GPC3), and MDA-MB231-vector (infected with the empty vector).

### Overexpression of ZEB1 in MDA-MB231-GPC3 cells

To overexpress ZEB1, 5 × 10^5^ MDA-MB231-GPC3 cells were transfected by lipofection employing Lipofectamine 2000 (Gibco LifeTechnologies, Carlsbad, CA, USA), with 3 μg of empty vector (pCDNAI/Amp) or ZEB1 expression vector (pCDNAI/ZEB1) [51]. Total extracts were used for immunoblots with anti ZEB1and anti E-Cadherin antibodies as was described in WB section.

### *In vitro* assays

#### Clonogenic capacity

800 monodispersed cells per well were seeded on 6-multiwell plates (Corning, Corning, NY, USA) in medium plus 10% FCS. Medium was changed every 72 h. After 7 days, plates were washed, fixed with 5% acetic acid in methanol and stained with crystal violet. The number of colonies (>10 cells) was counted under inverted microscope. Clonogenic capacity was defined as the percentage of cells able to grow as colonies under these conditions. The assay was performed in triplicate.

#### Anchorage-independent growth

To study if the genetically modified cells are able to form spheroids, 20,000 cells/well (pre-incubated or not for 1 h with 200 μg/ml of anti E-Cadherin polyclonal antibody, H-108 sc-7870 Santa Cruz Biotechnology, Dallas, TX, USA), or IgG (400 μg/ml as isotype control) were seeded in wells coated with 1.5% agar (Gibco LifeTechnologies, Carlsbad, CA, USA). After 7 days growing in suspension with medium plus 10% FCS (or 48 h for E-Cadherin antibody incubation), the spheroids were evaluated under inverted phase contrast microscope (Nikon, Eclipse TE2000-S). The experiment was done in triplicate.

#### Cell viability, death and apoptosis

Subconfluent monolayers were starved for 72 h. We performed Trypan blue exclusion assay to quantify viability (expressed as the percentage of living cells in relation to initially seeded cells). In addition, starved cells growing on coverslips were stained with 5 μg/ml of Höechst 33342 (Sigma-Aldrich, Saint Luis, MO, USA) and 5 mg/ml of propidium iodide (PI), for 15 min at 37°C. The percentage of cell death was calculated as the ratio of PI positive cells in relation to total cells stained with Höechst 33342. This relation was calculated in 10 random fields / coverslip. The experiment was done in triplicate.

Serum deprived MCF-7 sublines (72, 96, 120 and 144 h) as well as serum deprived MDA-MB231 sublines (18, 24, 48 and 72 h) were stained with 10 mg/ml acridine orange and 10 mg/ml ethidium bromide to assess apoptosis. Visualization was performed in a fluorescence microscope with 480 nm emissions (Nikon, Eclipse E400). Orange dyed cells were classified as apoptotic when they showed nuclear fragmentation (apoptotic bodies). The experiment was done in triplicate.

#### Cell migration

We performed a wound healing assay as described [28]. Briefly, cells were seeded in a 6-well plate and grown until confluence. Parallel wounds of about 400 μm width were created by scraping with a pipette tip. To assess the ability of the cells to migrate into the wound area, each wound was photographed in three random microscopic fields, and the initial area was measured using the Image-Pro Plus 6.0 program. After 17 h, the same fields were photographed and the migratory capacity was calculated as the difference of the cell-free area in each field. Experiments were carried out in triplicate.

### *In vivo* tumor xenograft model

All experiments were carried out using 2-month-old virgin female congenitally athymic nude mice (nu/nu) (25 g each) obtained from the Animal Care Area of the UNLP (La Plata National University, BA, Argentina). Experiments have been conducted in accordance with the ethical standards and according to national and international guidelines (NIH guide for the Care and Use of Laboratory Animals) and were approved by the Institutional Ethical Committee, Institute of Oncology “Ángel H. Roffo”, University of Buenos Aires (CD Res. 2012/11).

Non-anesthetized mice (from 5 to 10 animals / experimental group) were inoculated subcutaneously (s.c.) in the right flank with 7.5 × 10^6^ MCF-7 (-sh scramble and -sh3 C2) or with 7 × 10^6^ MDA-MB231 (-vector and -GPC3×2) cells in 0.2 ml serum-free RPMI 1640 medium. MCF-7 cells-bearing mice received an estrogen supplementation with s.c. pellets of 0.5 mg 17-β-estradiol, one week before inoculation. The two largest perpendicular diameters were recorded twice a week to evaluate tumor growth, and the volume was calculated. We did growth curves for each experimental group, from which we obtained the tumor growth rate (expressed as mm^3^/day).

Three month post-inoculation, mice were sacrificed and their tumors were dissected, fixed with 10% formalin and embedded in paraffin. 5 μm sections were stained with hematoxylin and eosin for histopathology. For invasiveness behavior analysis, macroscopic examination and histopathological study of serial sections of s.c. tumors were performed. Tumors were classified as invasive when cells migrated through s.c. skeletal muscle layer and reached the dermis.

To investigate the presence of spontaneous metastases, lungs were removed and fixed in Bouin's solution. Lungs were macroscopic and histologically examined. Two serial section separated 100 μm were selected to score for parenchymatous nodules and micrometastasis/metastasis tumor foci under a high-power microscope. Liver, kidney and spleen were also examined for the presence of metastatic nodules. Three independent experiments were performed.

### Statistical analysis

Data were presented as mean values ± standard deviation (SD), and statistical analyses were performed using Graph Pad InStat 3.0. The multiple comparisons of data were conducted by one way ANOVA test and Tuckey o Dunnett post-tests. Values of p<0.05 were considered statistically significant.
